# Evaluation and timing optimization of CT perfusion first pass analysis in comparison to maximum slope model in pancreatic adenocarcinoma

**DOI:** 10.1038/s41598-023-37381-w

**Published:** 2023-06-30

**Authors:** Neha Vats, Philipp Mayer, Franziska Kortes, Miriam Klauß, Lars Grenacher, Wolfram Stiller, Hans-Ulrich Kauczor, Stephan Skornitzke

**Affiliations:** 1 Clinic for Diagnostic and Interventional Radiology (DIR), Heidelberg University Hospital, Im Neuenheimer Feld 130.3, 69120 Heidelberg, Germany; 2 Radiology Rhein-Neckar, Bodelschwinghstraße 10, 68723 Schwetzingen, Germany; 3 Conradia Radiology and Medical Prevention, Conradia Radiologie München, Augustenstraße 115, 80798 Munich, Germany

**Keywords:** Cancer, Diagnostic markers, Pancreas

## Abstract

For implementation, performance evaluation and timing optimization of CT perfusion first pass analysis (FPA) by correlation with maximum slope model (MSM) in pancreatic adenocarcinoma, dynamic CT perfusion acquisitions of 34 time-points were performed in 16 pancreatic adenocarcinoma patients. Regions of interest were marked in both parenchyma and carcinoma. FPA, a low radiation exposure CT perfusion technique, was implemented. Blood flow (BF) perfusion maps were calculated using FPA and MSM. Pearson’s correlation between FPA and MSM was calculated at each evaluated time-point to determine optimum timing for FPA. Differences in BF between parenchyma and carcinoma were calculated. Average BF for MSM was 106.8 ± 41.5 ml/100 ml/min in parenchyma and 42.0 ± 24.8 ml/100 ml/min in carcinoma, respectively. For FPA, values ranged from 85.6 ± 37.5 ml/100 ml/min to 117.7 ± 44.5 ml/100 ml/min in parenchyma and from 27.3 ± 18.8 ml/100 ml/min to 39.5 ± 26.6 ml/100 ml/min in carcinoma, depending on acquisition timing. A significant difference (p value < 0.0001) between carcinoma and parenchyma was observed at all acquisition times based on FPA measurements. FPA shows high correlation with MSM (r > 0.90) and 94% reduction in the radiation dose compared to MSM. CT perfusion FPA, where the first scan is obtained after the arterial input function exceeds a threshold of 120 HU, followed by a second scan after 15.5–20.0 s, could be used as a potential imaging biomarker with low radiation exposure for diagnosing and evaluating pancreatic carcinoma in clinical practice, showing high correlation with MSM and the ability to differentiate between parenchyma and carcinoma.

## Introduction

Dynamic computed tomography (CT) perfusion is a functional imaging technique that provides the physiological information of the tissue perfusion along with the anatomical information non-invasively. It involves the acquisition of a baseline unenhanced CT image followed by the sequential acquisition of dynamic CT images after intravenous injection of an iodine-based contrast agent^1^. Early applications of CT perfusion were mainly focusing on brain, but some studies also performed dynamic contrast-enhanced CT acquisitions of the pancreas^2,3^. The physiological information obtained from dynamic CT turned out to be potentially important biomarkers for detection, diagnosis, and treatment planning of pancreatic tumors^4,5^. Nonetheless, the relatively high patient radiation exposure caused by the CT perfusion acquisition restricts its use in clinical practice. However, some studies made efforts in the past to reduce the radiation dose by decreasing the tube voltage, by using iterative reconstruction, or by evaluating dual-energy CT quantitative iodine concentration maps as a potential alternative to CT perfusion^6–8^. Although, CT perfusion is a commonly used functional imaging technique for pancreatic tumors, some studies have shown that magnetic resonance imaging (MRI) can also derive quantitative perfusion parameters, e.g. by intra-voxel incoherent motion (IVIM) or diffusion weighted MRI with similar performance in detection and diagnosis of pancreatic adenocarcinoma, without the radiation exposure of CT perfusion^4,9,10^. Regardless, CT perfusion is still the preferred imaging technique for quantitative perfusion measurements over IVIM and diffusion weighted MRI as it is less expensive, faster and more easily available^10^.

Over time, various mathematical models have been developed to calculate tissue perfusion measurements from dynamic CT acquisitions. These mathematical models translate the acquired tissue time attenuation curves (TACs) to the physiological perfusion parameters of the tissue in terms of blood flow (BF), blood volume, permeability, and mean transit time, etc.^1^. Studies have shown that the physiological information extracted using these models can help in improving diagnosis and treatment response assessment of pancreatic adenocarcinoma^5,11–13^. While the mathematical models stated above provide useful information for disease diagnosis, they also require multiple volume acquisitions to obtain the perfusion measurements, which leads to a relatively high radiation exposure.

On the other hand, the first-pass analysis (FPA) dynamic CT perfusion model uses only two acquisitions performed at two time points, thus, potentially drastically reducing the radiation dose by reducing the number of acquisitions^14,15^. The FPA technique has previously been implemented for myocardial perfusion measurements in phantom and animal studies^16–19^. Studies have also shown the potential of the FPA technique in diagnosis and detection of pulmonary diseases^14,15^. Hubbard et al. have implemented a timing optimization technique for the two volume scans required for the FPA perfusion measurement. They proposed a mathematical relationship between the contrast bolus injection time and contrast bolus time-to-peak for prospective acquisition of the two first-pass volume scans^20^. However, no studies have so far investigated the potential of the FPA technique in the diagnosis of pancreatic diseases.

Therefore, the objective of this study was to transfer the FPA technique to the pancreas and to investigate its potential for the accurate detection and diagnosis of pancreatic adenocarcinoma. To this end, the FPA technique was to be validated against MSM and the timing for a clinically applicable acquisition of the two volume scans required by FPA was to be optimized with regard to the pancreas.

## Methods

### Patient data

This retrospective study of prospectively acquired data^4^ evaluated the data of 23 patients (13 females, 10 males) with pancreatic adenocarcinoma. The inclusion criterion was detection of pancreatic adenocarcinoma in prior clinical examinations. The exclusion criteria were: patients with previous treatment of pancreatic adenocarcinoma, suspicion of hypervascular tumors, manifested hyperthyroidism, decreased kidney function, known hypersensitivity to iodinated contrast agent, inability to reproduce breathing technique, and/or denial of consent, as previously described^4^. Four patients were excluded based on histological diagnosis other than pancreatic adenocarcinoma and three due to excessive breathing motion during dynamic acquisition. Thus, out of the 23 patients, a total of 16 patients were included for the final analysis. The demographic information of these 16 patients has been summarized in Table 1.

**Table 1:** Demographic characteristics of patients used in this study.

Demographics information	
**Number of patients**	16
**Median age (interquartile range)**	61.5 (54–79 years)
**Sex**	
Female	9
Male	7
**T stage (tumor)**	
T1	1
T2	7
T3	4
Not available	4
**M stage (metastasis)**	
M0	12
Not available	4
**N stage (node)**	
N0	3
N1	3
N2	6
Not available	4
**Grading**	
G1	0
G2	8
G3	4
Not available	4
**Tumor location**	
Pancreatic head	14
Pancreatic body/tail	2
**ROI sizes [mean (mm**^2^**) ± SD]**	
**Circular ROI**	
Carcinoma (ROI2)	97.3 ± 47.9
Parenchyma (ROI3)	76.0 ± 47.4
**Polygonal ROI**	
Carcinoma (ROI4)	763.5 ± 576.0
Parenchyma (ROI5)	2508.3 ± 1105.8

### Image acquisition

The contrast-enhanced dynamic abdominal CT acquisitions were performed using a dual-source computed tomography scanner (SOMATOM Definition Flash; Siemens Healthineers, Germany). Before the dynamic acquisition, 80 ml of nonionic, iodinated contrast agent (Ultravist 370; Schering, Germany) were injected intravenously at a rate of 5.0 ml/s, followed by a saline solution (NaCl) bolus of 40 ml. After a delay of 13 s after the start of contrast-agent injection, the dynamic CT acquisition was started. The image acquisition consisted of 34 axial dynamic CT acquisitions with a temporal spacing of 1.5 s, over a period of 51 s (acquisition time 0.5 s, cycle time 1.5 s) at a tube voltage of 80 kV_p_/140 kV_p_ using automated tube current modulation with reference values of 270 mAs/104 mAs and a scan coverage of 19.2 mm. Image reconstruction was done using a soft tissue kernel B30f and 0.6 mm slice thickness.

### Region of interest (ROI) selection

**Figure 1:**
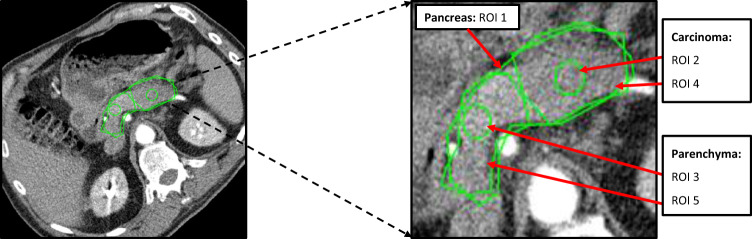
CT perfusion image with marked regions of interest (ROIs). CT image showing ROIs used for evaluation of a patient with pancreatic adenocarcinoma. Polygonal ROI1 outlines the whole pancreatic region. The carcinoma region has been marked by polygonal ROI4 and inside it, a small circular ROI2 has been marked to include carcinoma tissue only, excluding part of any other tissue or blood vessels. Similarly, the parenchyma region has been marked by a polygonal ROI5 and inside it, a small circular ROI3 has been placed to include parenchyma tissue only, excluding any other tissue or blood vessels.

ROIs were marked on the dynamic CT images by an experienced abdominal radiologist (F.K.). A reference image was selected from the dynamic CT images of a patient showing both non-neoplastic pancreatic parenchyma and carcinoma on the same slice. The whole pancreatic region was marked by a polygonal ROI (ROI1). A carcinoma region and a pancreatic parenchyma region, circumscribing each tissue, were marked by polygonal ROIs, named ROI4 and ROI5, respectively. Also, circular ROIs (as large as possible) were placed inside the polygonal ROIs to include only the respective tissue types with a high degree of confidence excluding any other tissue type or blood vessel, named ROI2 and ROI3, respectively. These ROIs were then copied from the reference image to all the images of respective patients. The arterial input function (AIF) was measured on the same images, i.e. in the abdominal aorta. Figure 1 shows the ROIs marked on a dynamic CT image of a pancreatic adenocarcinoma patient. An overview of ROI sizes is given in Table 1.

### Perfusion maps

An in-house developed software was used for motion correction^21^. Perfusion maps of blood flow [BF (ml/100 ml/min)] were calculated from the 80 kV_p_ images using two FPA approaches implemented in this study, as described in detail below. BF perfusion maps were also calculated using MSM^6^, and used as reference for validation of generated FPA perfusion maps.

The circular ROIs (ROI2 and ROI3) were used to assess the mean BF values with a high degree of confidence with respect to the respective tissue types. The polygonal ROIs (ROI4 and ROI5) were used to obtain the BF values for each voxel of the whole tissue region of the respective tissue type to perform voxel-based analysis.

### Maximum slope model (MSM)

MSM requires all 34 volume scans for perfusion measurement. MSM Perfusion (P_MSM_) is defined as the maximum upslope of the tissue time attenuation curves (dTAC/dt_max_), divided by the maximum of the AIF (AIF_max_) and tissue density (ρ_t_). In this study, MSM perfusion maps were generated using an in-house built software that implements MSM using a curve fitting model as described previously^6^.

### First pass analysis (FPA)

FPA proposes that the average perfusion (P_avg_) within a tissue compartment of interest is proportional to the first-pass entry of the contrast material mass into that compartment over time (dM_C_/dt), normalized by the incoming contrast material concentration (C_in_) and compartment tissue mass (M_t_), assuming no contrast material exits over the measurement duration, as shown in Eq. (1)^14^:

1 $${P}_{avg}={M}_{t}^{-1}{\left({C}_{in}^{-1}\frac{d{M}_{c}}{dt}\right)}_{avg}$$

dM_C_/dt is derived from the integrated change in TACs over time, while C_in_ is approximated from the AIF.

As P_avg_ is also proportional to the rate of contrast material concentration change within the compartment (i.e. the average change in tissue attenuation $(\Delta H{U}_{avg}$) over time), the voxel-by-voxel concentration change ($\Delta HU$) is used to derive voxel-by-voxel perfusion (P_FPA_), as shown in Eq. (2)^14^:

2 $${P}_{FPA}={P}_{avg}\frac{\Delta HU}{{\Delta HU}_{avg}}$$

**Figure 2:**
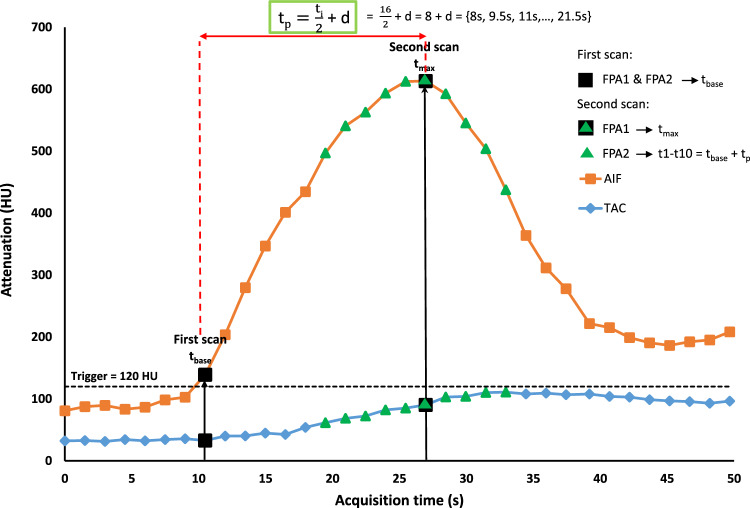
First pass analysis (FPA) acquisition protocol. A tissue attenuation curve (TAC) of an ROI placed inside pancreatic parenchyma and arterial input function (AIF) in the abdominal aorta. All 34 scans of a dynamic CT acquisition as used in MSM are represented by squared boxes on the curves. Two black colored boxes represent the two volume acquisitions required in FPA: the first scan is marked after crossing the threshold of 120 HU (t_base_). For FPA1, the second scan is marked at the peak of the AIF (t_max_). For FPA2, the second scan is taken ‘t_p_’ seconds after the first scan, as marked by triangles on the curve from t1–t10. *D* dispersion delay, *t*_*base*_ time of baseline acquisition of AIF, *t*_*i*_ time taken for contrast injection, *t*_*max*_ time of peak acquisition of AIF, *t*_*p*_ time at temporal center of the bolus.

FPA only requires two CT volume scans, where the first volume scan should be acquired at the baseline (t_base_) of the AIF curve while the second one is to be acquired at the peak (t_max_) of the AIF as suggested by Hubbard et al. in their study from 2018^14^, shown in Fig. 2. Adapting the proposed acquisitions to the pancreas, the first volume scan at t_base_ was selected when the AIF just exceeds 120 Hounsfield units (HU)^22^. However, in clinical practice the timing for the second volume scan at t_max_ is not known before acquisition. Therefore, an approach to approximate the timing of t_max_ in clinical practice was implemented and compared to the theoretical optimum of an acquisition at t_max_. In this study, these two volume scans were retrospectively selected from the 34 dynamically acquired volume scans based on the calculated timing.

#### FPA1

In the first FPA implementation (FPA1), the first volume scan was selected as the scan where the contrast agent bolus exceeds the threshold of 120 HU in the abdominal aorta, i.e. t_base_. For the second scan, the acquisition time where the AIF reached its maximum (t_max_) was retrospectively determined, as per the theoretical definition stated by Hubbard et al.^14^. Thus, volume scans selected at t_base_ and t_max_ were used to calculate FPA1 BF perfusion maps. However, this FPA technique is only theoretically possible, as the peak of AIF (t_max_) is not known a-priori.

#### FPA2

In the second FPA implementation (FPA2), the scan at t_base_ was selected as the first volume scan, while the timing to select second scan was adapted from Hubbard et al.^20^: based on the studies by Garcia et al.^23^ and Han et al.^24^, Hubbard et al. proposed that the temporal center of the contrast agent bolus (t_p_) maintains the highest contrast agent concentration and peak enhancement. The timing of this temporal center can be approximated from one half the injection time ($\frac{{t}_{i}}{2}$) plus a fixed dispersion delay (d), as described by Eq. (3) and displayed in Fig. 2:

3 $${t}_{p}=\frac{{t}_{i}}{2}+d$$

where t_i_ is the duration of contrast agent injection, i.e. 16 s in our study and thus $\frac{{t}_{i}}{2}$ = 8 s. To determine a suitable dispersion delay, perfusion maps were generated by using ten different values for d, resulting in ten different acquisition timings for the second scan. Compared to the myocardium, a larger degree of dispersion can be expected for the pancreas, thus ten acquisition timings (t1–t10) were evaluated compared to five different timings used by Hubbard et al.^20^. The dispersion delay (d) was iteratively increased from 0 to 13.5 s with an interval of 1.5 s, resulting in range of t_p_ values = {8 s, 9.5 s, 11 s… 21.5 s}. Thus, the scan at time t_p_ after the first scan was retrospectively selected as the second scan. BF perfusion maps were obtained at ten different acquisition times (t1–t10) using FPA2.

### Evaluation

Mean ± standard deviation (SD) were calculated for BF perfusion values obtained using FPA1, FPA2, and, MSM for both the circular ROIs (ROI2 and ROI3) and the polygonal ROIs (ROI4 and ROI5) for respective tissue regions. BF values calculated using circular ROIs provided higher degree of confidence in the respective tissue types, as no other tissue or blood vessels were included, unlike polygonal ROIs. Polygonal ROIs were also used for voxel-based evaluation of calculated BF maps.

### Statistical analysis

Statistical analysis was performed using Excel 2016 (Microsoft Corporation; USA), SAS software (version 9.4, SAS Institute; USA) and MATLAB R2022a (MathWorks; USA). Mean and standard deviation values were calculated for the BF for ROI2–ROI5. Differences in BF between carcinoma and parenchyma tissue were assessed using student’s t-test for the mean values of ROI2 and ROI3 as well as for all voxels included in ROI4 and ROI5.

Correlation between FPA and MSM measurements was evaluated by calculating Pearson’s correlation coefficient (r). Correlation was assessed for the mean values measured in the circular ROIs (ROI2 and ROI3) and also, for all voxels in the polygonal ROIs (ROI4 and ROI5).

Coefficients of variation (COV) were calculated to measure the sensitivity of perfusion values to variations in acquisition timing when using FPA2. Box-whisker plots were generated to compare the perfusion values at varying acquisition times and for different tissue regions.

### Radiation exposure and acquisition time

The radiation dose to the patients was estimated by multiplication of radiation exposure [calculated in terms of dose-length product (DLP)] with the conversion factor for abdominal CT examinations (0.0153 mSv/mGy·cm), where DLP is the product of the volumetric CT dose index (CTDI_vol_) with scan length.

The acquisition time (s) was estimated by the total amount of time taken for the complete acquisition of all the dynamic CT images. The total acquisition time for FPA was estimated from the start of the bolus monitoring to the last time point of the optimum time range i.e. t9.

### Ethical approval

The study protocol was approved by the local ethics committee of the University Hospital Heidelberg and conducted in accordance with the ethical standards of the World Medical Association (Declaration of Helsinki). All subjects provided written informed consent before undergoing CT scanning for study participation.

## Results

### MSM

The mean ± SD of MSM BF values obtained from the circular ROIs were 42.0 ± 24.8 ml/100 ml/min for carcinoma (ROI2) and 106.8 ± 41.5 ml/100 ml/min for parenchyma (ROI3), respectively (Table 2). Also, the mean ± SD of MSM BF values obtained from the polygonal ROIs were 53.7 ± 23.9 ml/100 ml/min for carcinoma (ROI4) and 91.2 ± 37.1 ml/100 ml/min for parenchyma (ROI5), respectively (Table 3).

**Table 2:** Mean ± SD blood flow (BF) values of carcinoma (ROI2) and parenchyma (ROI3) tissue for circular ROI using FPA1, FPA2 at all ten acquisition times and MSM.

Tissue type	FPA1	FPA2										MSM
	t_max_	t1	t2	t3	t4	t5	t6	t7	t8	t9	t10	
Circular ROI blood flow (ml/100 ml/min) measurement												
Carcinoma	30.3 ± 19.2	36.7 ± 20.8	34.4 ± 17.6	32.8 ± 15.6	29.9 ± 17.3	29.8 ± 16.1	31.9 ± 20.0	27.3 ± 18.8	32.2 ± 21.0	37.9 ± 23.0	39.5 ± 26.6	42.0 ± 24.8
Parenchyma	89.0 ± 31.7	105.4 ± 56.7	102.7 ± 56.1	99.6 ± 45.5	92.1 ± 32.1	91.4 ± 40.2	85.6 ± 37.5	89.6 ± 31.3	98.2 ± 36.8	102.0 ± 41.5	117.7 ± 44.5	106.8 ± 41.5
T-test (p value)	< 0.0001	0.0001	< 0.0001	< 0.0001	< 0.0001	< 0.0001	< 0.0001	< 0.0001	< 0.0001	< 0.0001	< 0.0001	< 0.0001
Pearson’s correlation (r)	0.95	0.82	0.86	0.87	0.93*	0.94*	0.91*	0.93*	0.93*	0.92*	0.90	

Circular ROI were used to indicate the respective tissues with a high degree of confidence. Student’s t-test p value shows a significant difference between carcinoma and the parenchyma tissue. Correlation values marked with asterisk show the range of acquisition time (t4–t9) with the highest correlation (r > 0.90) between FPA2 and MSM.

**Table 3:** Mean ± SD blood flow (BF) values of carcinoma (ROI4) and parenchyma (ROI5) tissue for polygonal ROI using FPA1, FPA2 at all ten acquisition times and MSM.

Tissue type	FPA1	FPA2										MSM
	t_max_	t1	t2	t3	t4	t5	t6	t7	t8	t9	t10	
Polygonal ROI blood flow (ml/100 ml/min) measurement												
Carcinoma	42.3 ± 16.4	49.5 ± 18.0	44.2 ± 15.7	46.9 ± 18.3	44.2 ± 18.7	39.6 ± 14.7	42.5 ± 16.3	41.9 ± 16.9	44.7 ± 17.8	51.4 ± 19.4	54.1 ± 22.5	53.7 ± 23.9
Parenchyma	74.6 ± 29.6	82.6 ± 34.7	84.9 ± 34.8	80.5 ± 32.3	78.5 ± 31.4	76.0 ± 32.7	73.7 ± 30.8	76.1 ± 28.8	80.5 ± 32.5	87.8 ± 34.9	97.9 ± 38.9	91.2 ± 37.1
T-test (p value)	< 0.0001	< 0.0001	< 0.0001	< 0.0001	< 0.0001	< 0.0001	< 0.0001	< 0.0001	< 0.0001	< 0.0001	< 0.0001	< 0.0001
Pearson’s correlation (r)	0.66	0.38	0.55	0.57	0.56	0.66	0.72*	0.75*	0.73*	0.72*	0.67	

Polygonal ROIs were used to circumscribe respective tissues for voxel-by-voxel analysis. Student’s t-test p value for voxel values in polygonal ROIs shows a significant difference between carcinoma and parenchyma at all ten acquisition times. Correlation values marked with an asterisk show the range of acquisition time (t6–t9) with the highest correlation (r > 0.7) between FPA2 and MSM for each voxel value in the polygonal ROI.

### FPA1

**Figure 3:**
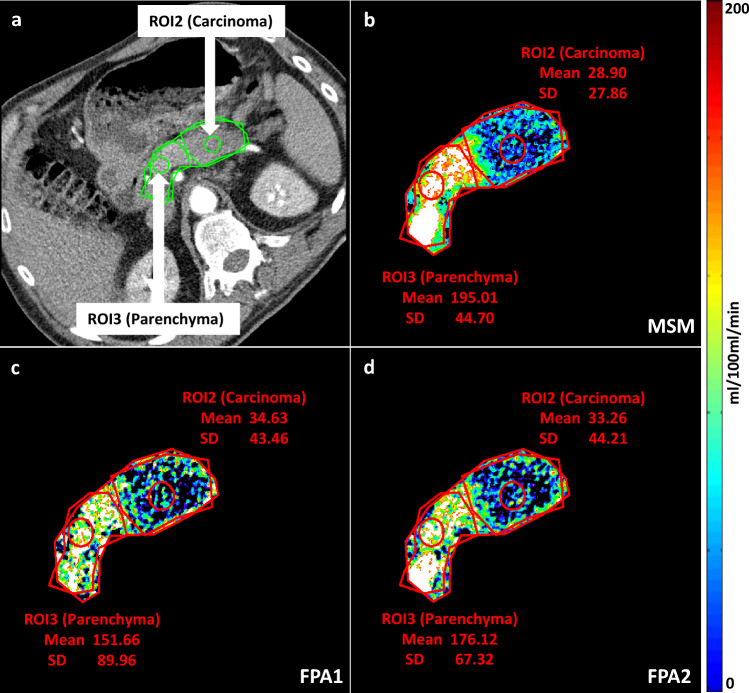
First pass analysis (FPA) and maximum slope model (MSM) perfusion maps. Conventional dynamic CT image (**a**), MSM perfusion map (**b**), FPA1 perfusion map at t_max_ (**c**) and FPA2 perfusion map at t9 (**d**) are shown for an adenocarcinoma patient. The blood flow (BF) perfusion maps show mean and SD values for circular ROI2 (carcinoma) and ROI3 (parenchyma). Note the higher mean BF of parenchyma tissue as compared to carcinoma for FPA1, FPA2, and MSM. Mean values are comparable between FPA1, FPA2, and MSM for the corresponding tissue regions.

An example of BF maps obtained using FPA1 and MSM is shown in Fig. 3. The mean ± SD of BF values were observed to be slightly lower for FPA1 as compared to MSM: 30.3 ± 19.2 ml/100 ml/min for carcinoma (circular ROI2), and 89.0 ± 31.7 ml/100 ml/min for parenchyma (circular ROI3) as mentioned in Table 2, and 42.3 ± 16.4 ml/100 ml/min for carcinoma (polygonal ROI4), and 74.6 ± 29.6 ml/100 ml/min for parenchyma (polygonal ROI5) as mentioned in Table 3.

### FPA2

An example of BF maps obtained using FPA2 is shown in Fig. 3. The mean ± SD of BF values calculated for the carcinoma (circular ROI2) and the parenchyma (circular ROI3) tissue using at all ten time-points for FPA2 are stated in Table 2. Also, mean ± SD BF values calculated for whole carcinoma tissue (polygonal ROI4) and whole parenchyma tissue (polygonal ROI5) using at all ten time-points for FPA2 are stated in Table 3.

**Figure 4:**
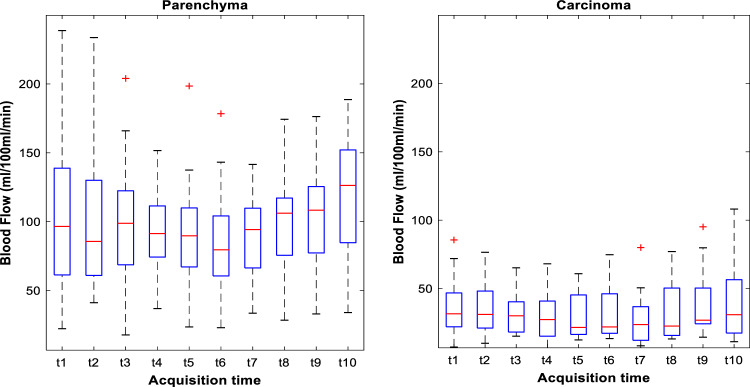
Box plot analysis at different acquisition timings. Box plots for FPA2 blood flow (BF) measurements at the investigated acquisition times for both carcinoma (circular ROI2) and parenchyma (circular ROI3) tissue.

BF measurements obtained using FPA2 were comparable to FPA1 measurements. The box-plot analysis for BF values using circular ROIs at different acquisition timings for both carcinoma and parenchyma tissue are shown in Fig. 4. The box plot shows that the acquisition range from t4 to t9 exhibits reduced spread in the BF values compared to the other acquisitions, while parenchyma shows overall reduced spread of BF values compared to carcinoma. Overall, the sensitivity of BF values to acquisition timing is low as demonstrated by low COV values of 11.6% for carcinoma and 9.5% for parenchyma between multiple time points. COV between time points for the polygonal ROIs was further reduced to 9.9% for carcinoma and 8.7% for parenchyma, respectively.

### Correlation between FPA and MSM (FPA1-MSM and FPA2-MSM)

**Figure 5:**
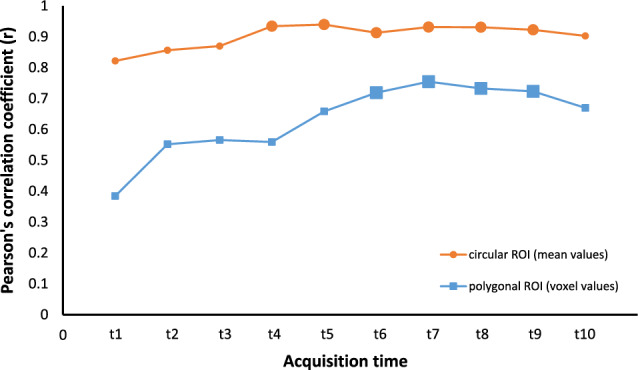
Correlation curves between FPA2 and MSM. Pearson’s correlation curves between FPA2 and MSM at the investigated acquisition times (t1–t10) for carcinoma and parenchyma tissue combined. Acquisitions with a high correlation are highlighted by bold markers.

Correlation between mean values of BF measurements in the high degree of confidence region, i.e. circular ROIs (ROI2 and ROI3), was high between FPA1 and MSM (r = 0.95) as well as between FPA2 and MSM (r = 0.82–0.94, depending on acquisition time). Highest correlations between FPA2 and MSM were found for acquisitions at t4–t9 (r = 0.91–0.94), respectively, as shown in Table 2. Also, BF measurements of each individual voxel in the polygonal ROIs (ROI4 and ROI5) show high correlation^25^ between FPA1 and MSM (r = 0.66), and between FPA2 and MSM (r = 0.38–0.75). Highest correlations between FPA2 and MSM were found for acquisitions between t6 and t9 (r = 0.72–0.75), as shown in Table 3. Figure 5 shows the correlation curves between FPA2 and MSM for both mean values and individual voxels with the highlighted optimum time range for FPA2.

### Differences between tissue types

Differences between mean values of circular ROIs in parenchyma and carcinoma (ROI2 and ROI3) were significant for FPA1, FPA2, and MSM (p < 0.0001). Differences between voxel values in polygonal ROIs (ROI4 and ROI5) were also significant for FPA1, FPA2, and MSM (p < 0.0001).

### Radiation exposure and acquisition time

**Figure 6:**
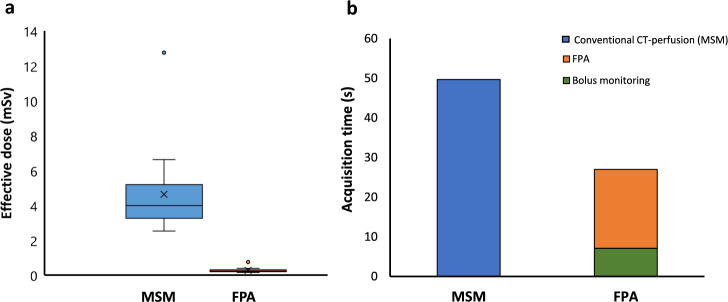
Comparison of effective dose and scan time between MSM and FPA. Box plot (**a**) and bar graph (**b**) show the patient radiation exposure, indicated by effective dose (mSv), and total acquisition time (s) of MSM and FPA, respectively.

For an FPA acquisition using only two volume scans, effective radiation dose to the patient was 0.27 ± 0.14 mSv and total acquisition time was 27.02 s, as compared to 4.64 ± 2.32 mSv radiation dose and 49.68 s acquisition time for a conventional CT perfusion acquisition at a tube voltage of 80 kV_p_, respectively. Figure 6 shows a comparison of effective radiation dose and scan time between MSM and FPA.

## Discussion

Dynamic CT perfusion is an important potential biomarker for improved detection and diagnosis of pancreatic tumors, but the use of multiple volume acquisitions leads to increased patient radiation exposure, restricting its application in clinical practice^4,5^. In this study, an FPA perfusion technique using only two volume acquisitions was implemented. The reduction in the number of volume acquisitions reduces the radiation dose, thus, overcoming one of the limitations of dynamic CT perfusion. FPA showed high correlation with the dynamic CT perfusion measurements in pancreatic adenocarcinoma. This implies that the low radiation dose FPA technique could be used as an imaging biomarker for perfusion measurements of pancreatic tumors in clinical practice in the near future.

The first FPA approach implemented, i.e. FPA1, showed the maximum correlation (r = 0.95) with MSM. However, FPA1 requires knowledge of the exact acquisition time when the AIF reaches its maximum (t_max_), which is not available before the acquisition. Therefore, regardless of the high correlation to MSM, implementation of FPA1 is not suitable to be considered for clinical applications.

The second FPA approach, i.e. FPA2, also showed high correlation^25^ (r = 0.82–0.94, depending on acquisition time) with MSM for a range of acquisition times and only requires contrast agent injection information to estimate the acquisition timing of the second volume scan, which is available prior to the scan. This makes it easier to calculate the acquisition time for the second volume scan, overcoming the limitation of FPA1 and making an implementation of FPA2 possible in clinical practice. A small change in the acquisition time within the optimum time range (t4–t9) yields only small differences in BF, therefore, a high correlation to MSM of r = 0.91–0.94 can be achieved depending upon the selection of acquisition time within the optimum time range. The results showed that one half the injection time plus a fixed dispersion delay provides a high correlation coefficient value at that time, as stated by Hubbard et al.^20^. The determined timings for FPA2 in this study also yield results that are almost as good as the theoretical optimum implemented by FPA1. Thus, FPA2 seems to be a practical solution to the theoretical concept of FPA. However, the dispersion delays providing high correlation with MSM in this study did not agree with the optimum dispersion delay determined by Hubbard et al.: the optimum dispersion delay ‘d’ as reported by Hubbard et al.^20^ was 1 s, whereas the optimum dispersion delay obtained in this study ranges between 7.5 and 12.0 s. The reason for this difference in dispersion delays might be the difference in organs, i.e. pancreas versus myocardium. Thus, calculation of different dispersion delays based on the investigated organ or anatomical region should be considered. Also, the difference in the FPA2 perfusion values was small when varying acquisition time, as shown by the small COV. Thus, a small change in the acquisition timing will not create much difference in the perfusion measurements and accuracy of the disease diagnosis, suggesting that FPA2 is robust to changes in the acquisition timing or the patient’s circulatory function. Thus, FPA perfusion measurement in the optimum acquisition time range of t6–t9 might improve diagnosis of pancreatic carcinoma. Hence, the FPA2 approach seems feasible for application in clinical practice for assessment of pancreatic diseases.

For clinical application of FPA2, first a nonionic, iodinated contrast agent is to be injected intravenously, followed by a saline solution (NaCl) bolus^14^. For the pancreas, the first volume scan of FPA is to be acquired after the contrast agent bolus reaches a threshold of 120 HU in the abdominal aorta, which can be achieved by bolus tracking. The second volume scan is to be acquired after a delay of 15.5–20.0 s, based on the results of the current study.

Theoretically, implementation of FPA using only two volume scans reduces the total scan time from 49.68 s in conventional CT perfusion to 27.02 s in the proposed technique i.e. a potential reduction of up to 45.62% in the scan time, thus reducing the motion artifacts present in other CT perfusion measurements due to holding breath for a longer scan duration. Additionally, FPA limits the radiation exposure to the patient, by using only two volume acquisitions as compared to multiple volume scans required by conventional CT perfusion. In this retrospective study, the two volume scans would have been achieved at an effective radiation dose of only 0.27 ± 0.14 mSv as compared to 4.64 ± 2.32 mSv radiation dose required by dynamic CT perfusion, i.e. a potential reduction of up to 94% in the effective radiation dose, not including the effective dose of the bolus tracking. Considering the reduction in radiation exposure and scan time of the proposed FPA2 compared to conventional CT perfusion, the technique could be established as a potential imaging biomarker for the quantitative detection, disease diagnosis and treatment planning of the pancreatic tumors in clinical practice.

However, despite FPA being a potential alternative to dynamic CT perfusion, this study has some limitations. Firstly, the technique needs to be implemented on larger datasets for better reliability as this study was limited to 16 patients only. Also, this study is a retrospective study and prospective validation of the proposed acquisition time seems necessary. Furthermore, no qualitative evaluation has been performed and the diagnostic use of FPA perfusion maps has not been investigated, which is necessary for further validation. Thus, further investigation in this area is required prior to actual application in clinical practice.

## Conclusion

The proposed FPA approach using only two volume scans, where the first scan is acquired after the threshold of 120 HU is reached in the abdominal aorta, followed by the second scan acquired 15.5–20.0 s after the first scan, has the potential to provide an alternative imaging biomarker to conventional CT perfusion for pancreas. FPA shows the ability to differentiate pancreatic adenocarcinoma from parenchyma with high correlation to MSM. FPA could also allow for a large decrease in patient radiation exposure and scan time as compared to conventional CT perfusion for evaluating pancreatic diseases.

## Data Availability

The datasets analyzed during the current study are not publicly available due to ethical reasons but are available from the corresponding author on reasonable request.

## Abbreviations

AIF: Arterial input function
AIF_max_: Maximum of AIF
BF: Blood flow
C_in_: Incoming contrast material concentration
COV: Coefficient of variation
CT: Computed tomography
CTDI_vol_: Volumetric CT dose index
d: Dispersion delay
DLP: Dose-length product
dM_C_/dt: Contrast material mass entering a compartment over time
dTAC/dt_max_: Maximum upslope of tissue time attenuation curve
ΔHU_avg_: Average change in tissue attenuation over time
∆HU: Voxel-by-voxel concentration change
FPA: First pass analysis
HU: Hounsfield Unit
IVIM: Intra-voxel incoherent motion
MRI: Magnetic resonance imaging
MSM: Maximum slope model
M_t_: Compartment tissue mass
P_avg_: Average FPA perfusion
P_FPA_: Voxel-by-voxel FPA perfusion
P_MSM_: MSM perfusion
R: Pearson’s correlation coefficient
ROI: Region of interest
ρ_t_: Tissue density
SD: Standard deviation
TAC: Tissue attenuation curve
t_base_: Time of baseline acquisition of AIF
t_i_: Time taken for contrast injection
t_max_: Time of peak acquisition of AIF
t_p_: Time at temporal center of the bolus
